# Regulation of the Signal-Dependent E Protein HEBAlt Through a YYY Motif Is Required for Progression Through T Cell Development

**DOI:** 10.3389/fimmu.2022.848577

**Published:** 2022-08-03

**Authors:** Kogulan Yoganathan, Anqi Yan, Juliana Rocha, Ashton Trotman-Grant, Mahmood Mohtashami, Lisa Wells, Juan Carlos Zúñiga-Pflücker, Michele K. Anderson

**Affiliations:** ^1^ Biological Sciences, Sunnybrook Research Institute, Toronto, ON, Canada; ^2^ Department of Immunology, University of Toronto, Toronto, ON, Canada

**Keywords:** T cell development, HEB, TCF12, gene expression, signal transduction

## Abstract

The E protein transcription factors E2A and HEB are critical for many developmental processes, including T cell development. We have shown that the *Tcf12* locus gives rise to two distinct HEB proteins, with alternative (HEBAlt) and canonical (HEBCan) N-terminal domains, which are co-expressed during early T cell development. While the functional domains of HEBCan have been well studied, the nature of the HEBAlt-specific (Alt) domain has been obscure. Here we provide compelling evidence that the Alt domain provides a site for the molecular integration of cytokine signaling and E protein activity. Our results indicate that phosphorylation of a unique YYY motif in the Alt domain increases HEBAlt activity by 10-fold, and that this increase is dependent on Janus kinase activity. To enable *in vivo* studies of HEBAlt in the T cell context, we generated ALT-Tg mice, which can be induced to express a HA-tagged HEBAlt coding cassette in the presence of Cre recombinases. Analysis of ALT-Tg mice on the Vav-iCre background revealed a minor change in the ratio of ISP cells to CD8+ SP cells, and a mild shift in the ratio of T cells to B cells in the spleen, but otherwise the thymus, spleen, and bone marrow lymphocyte subsets were comparable at steady state. However, kinetic analysis of T cell development in OP9-DL4 co-cultures revealed a delay in early T cell development and a partial block at the DN to DP transition when HEBAlt levels or activity were increased. We also observed that HEBCan and HEBAlt displayed significant differences in protein stability that were resolved in the thymocyte context. Finally, a proteomic screen identified STAT1 and Xpo1 as potential members of HEBAlt-containing complexes in thymocytes, consistent with JAK-induced activation of HEBAlt accompanied by translocation to the nucleus. Thus, our results show that the Alt domain confers access to multiple layers of post-translational control to HEBAlt that are not available to HEBCan, and thus may serve as a rheostat to tune E protein activity levels as cells move through different thymic signaling environments during T cell development.

## Introduction

T cells act as a central organizing hub for adaptive immune responses and provide a first line of innate defense in barrier tissues. These roles are distributed among distinct T cell subsets, which acquire their core functions during T cell development in the thymus. T cell development occurs in the thymus through a series of intermediates that give rise to αβ and γδ T cells. These cells arise from T-lineage committed progenitors known as double negative (DN; CD4-CD8-) cells. DN cells can be further subdivided into successive developmental stages using the markers CD44 and CD25: DN1 (CD44^+^CD25^-^), DN2 (CD44^+^CD25^+^), DN3 (CD44^-^CD25^+^), and DN4 (CD44^-^CD25^-^) (1). Successful rearrangement of TCRβ, expression of TCR signaling components, and assembly of a pre-T cell receptor (pre-TCR) complex allows passage through “β-selection” into the αβ-T lineage (2). β-selected cells downregulate CD25 to become DN4 cells. DN4 cells undergo rapid proliferation and upregulate CD8^+^ to become immature single positive (ISP) cells. This is followed by upregulation of CD4 to generate double positive (DP; CD4^+^CD8^+^) thymocytes (3). DP cells become quiescent as they commence TCRα rearrangement. Upon αβ TCR signaling, DP cells can differentiate into either conventional CD4^+^ or CD8^+^ single positive (SP) cells through a series of intermediate stages that can be followed by expression of CD69 and CD24.

The E protein transcription factors encoded by the *Tcf12* (HEB) and the *Tcf3* (E2A) loci are essential regulators of T cell development. Each major developmental transition that occurs during thymic T cell development is dependent on interactions between E protein transcription factors and their antagonist Id3 (4–8). Prior to β-selection, E proteins directly upregulate the expression of proteins involved in pre-TCR signaling, including pTα, TCRβ, CD3, Lck, LAT, and RAG1/2 (9–12). Pre-TCR, γδ TCR, and αβ TCR signaling lead to the transient upregulation of Id3, which halts E protein activity (13, 14). After T lineage commitment and passage through β-selection, E proteins can regulate gene products that are not expressed at the DN stage, including CD4, TCRα, and Rorγt (7, 15–17). Thus, regulation of different suites of T cell genes at different stages of development is a key feature of E protein activity during T cell development, as has also been observed for GATA3 and Runx factors (18, 19).

HEB and E2A knockout models have provided considerable insight into the roles of these factors at different stages of thymocyte development. HEB deficiency leads to defects in fetal γδ and αβ T cell development (20, 21). HEB disruption also results in a partial block at the DN3 to DN4 transition, an accumulation of ISP cells, and a decrease in CD4 T cells (22, 23). Deletion of E2A results in a partial block at the DN1 to DN2 transition, breakthrough to the DP stage in the absence of pre-TCR or γδTCR signals, an increase in SPs, and leukemic transformation of T cell progenitors (24, 25). Conditional deletion of both HEB and E2A with Lck-Cre in DN thymocytes resulted in the emergence of a rapidly cycling population of IL-7R dependent DN2-like cells (26). These studies and others led to the concept of E proteins as “gatekeepers” that prevent inappropriate differentiation and proliferation prior to receiving pre-TCR, γδTCR, or αβTCR signals (27).

**Figure 1:**
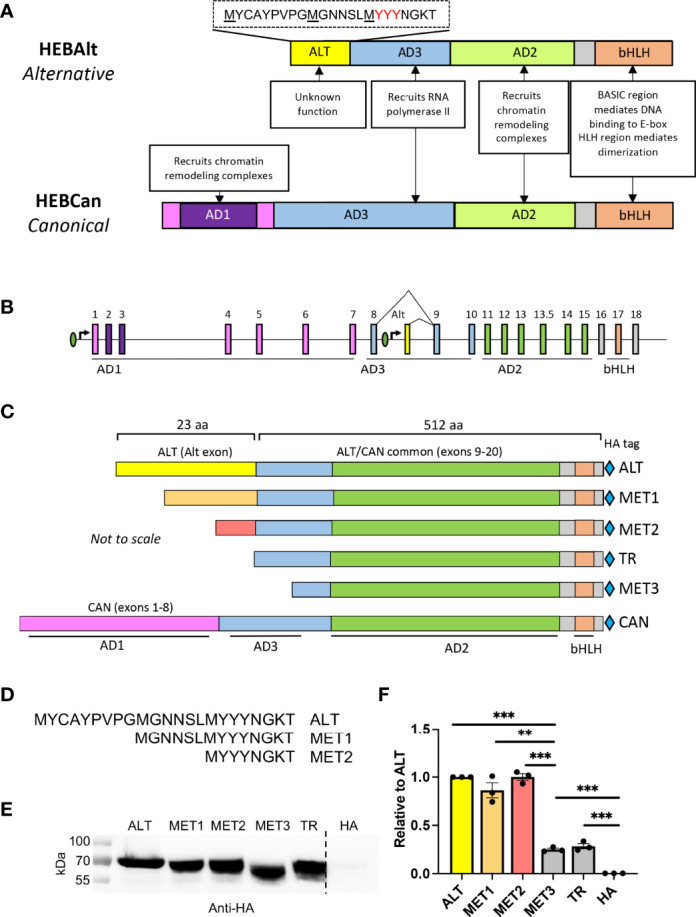
The Alt domain is required for HEBAlt transcriptional activity. **(A)** Diagram of HEBAlt (alternative) and HEBCan (canonical) variant domain structures and functions. AD=activation domain, bHLH = basic DNA binding and helix-loop-helix dimerization. ALT = domain specific to HEBAlt, with amino acid sequence shown above it. **(B)** Structure of the *TCF12* gene locus that encodes both HEBCan and HEBAlt by alternative transcriptional initiation and splicing. Introns = straight line, exons = boxes, promoters = ovals. Exons encoding the functional domains are indicated under the locus and in colors corresponding to **(A)**. **(C)** Domain structures of HEBAlt WT (ALT), MET1, MET2, TR, MET3, and HEBCan (CAN). Domains are color coded to match **(A, B)**. Light blue diamonds = HA epitope tag. **(D)** Amino acid sequence of the wildtype (ALT) and mutated Alt (MET1, MET2) domains. MET3 and TR lack the ALT domain entirely. **(E)** Western blot showing protein expression of wildtype and mutant HEBAlt construct expression as detected by anti-HA. HA indicates HA vector-only control **(F)**. Dual luciferase assay on cells co-transfected with HA-tagged ALT, MET1, MET2, MET3, or TR, plus 8X E box Firefly luciferase and Renilla luciferase constructs. The Y-axis depicts relative luciferase units (RLU) of Firefly to Renilla values, normalized to ALT. ***P ≤ 0.0001, **P ≤ 0.001, ns = non-significant. Note that in **(E)**, some lanes from the same gel are depicted separately, as they were cut and pasted back together from the original image to exclude irrelevant data. None of the domain or construct diagrams are to scale.

HEB and E2A function as homodimers and heterodimers, but how dimer composition affects target gene expression is poorly understood. In DN thymocytes, two variants of HEB are expressed: HEBAlt (alternative) and HEBCan (canonical) (28). HEBAlt mRNA is downregulated at the DN3 to DN4 transition, leaving HEBCan and E2A as the main E proteins in DP and SP cells (28, 29). HEBCan and E2A share a conserved domain structure, including three activation domains (AD1, AD2, AD3), which interact with other transcriptional regulators, and a basic helix-loop-helix (bHLH) DNA binding and dimerization domain (30–32) (**Figure 1A**; **Figure S1**). HEBAlt includes AD2 and the bHLH domain and lacks AD1. The N-terminus of HEBAlt encodes a 23 amino acid “Alt” domain that is excluded from HEBCan. This configuration arises from the location of the Alt exon between exons 8 and 9 of the *Tcf12* gene locus (**Figure 1B**). Additionally, the AD3 domain spans exons 8 and 9, resulting in the absence of the first half of AD3 in HEBAlt.

HEBAlt and HEBCan are expressed from distinct transcriptional start sites, allowing differential regulation of mRNA expression (**Figure 1B**). HEBCan is expressed throughout T cell development, peaking at the DP stage, whereas HEBAlt mRNA is restricted to DN2 and DN3 cells (28). Thus, differences between HEBAlt and HEBCan could factor into the switching of E protein target genes at the DN to DP transition. Our previous work using retroviral vectors and *in vitro* differentiation systems showed that HEBAlt could enhance entry of uncommitted progenitors into the T-lineage, whereas HEBCan did not (28, 33). HEBAlt could also uniquely restrict B and myeloid cell development, and recruit committed myeloid precursors into the T cell lineage (21, 33, 34). By contrast, overexpression of HEBCan inhibited T cell development, consistent with its role as a gatekeeper. Whether HEBAlt participates in the gatekeeping process has not been resolved, and the function of the Alt domain remains unclear.

In this study, we generated HEBAlt mutant constructs and evaluated their ability to induce transcriptional activation using promoter-reporter luciferase assays. Our results identified a unique triple tyrosine (YYY) motif within the Alt domain that plays a role in the magnitude of HEBAlt-mediated transcriptional activation. We also showed that YYY-mediated elevation of HEBAlt activity is dependent on JAK (Janus tyrosine kinase) activity, and that the YYY motif can be phosphorylated. Furthermore, using an HEBAlt transgenic mouse model, we observed that uncontrolled HEBAlt activity inhibited progress through T cell development, and that HEBAlt protein stability is dependent on cell context. Our results indicate that HEBAlt activity is tightly regulated at the post-translational level, and that disruption of this regulation interferes with T cell development.

## Methods

### Mice

The generation of Rosa26-loxP-stop-loxP-HEBAlt-HA (ALT-Tg) mice was performed by Ingenious Targeting Laboratories (Ronkonkoma, NY). Hybrid (129/SvEv x C57BL/6) embryonic stem cells (ESCs) were targeted and microinjected into C57BL/6 blastocysts. Resulting chimeras were mated to WT C57BL/6N mice to generate F1 heterozygous offspring, and those with germline integration were backcrossed for six generations with C57BL/6 mice to fix them on the C57BL/6 background. To induce expression in all hematopoietic cells, ALT-Tg mice were crossed to Vav-iCre mice (Jax; 008610)91. All experiments were conducted using 6-8 wk old littermate controls. Mice were maintained at the Sunnybrook Research Institute and all protocols were approved by the Animal Care Committee.

### Construct Generation

HEBAlt (NM_001253864.1) and HEBCan (NM_011544.3) cDNAs were cloned into pCMV-HA or pCMV-myc expression vectors using the KpnI and SalI cloning sites. Site-directed mutagenesis performed on the pCMV-HEBAlt-HA plasmid to generate the mutant constructs using the Agilent QuikChange Site-Directed Mutagenesis Kit or Q5^®^ Site-Directed Mutagenesis Kit. Mutations were confirmed by cloning the inserts into Top10 Competent E. coli cells, followed by plasmid DNA extraction and Sanger Sequencing (TCAG; SickKids, Toronto, Canada).

### Cell Culture

Cells lines were cultured under standard conditions at 37°C with 5% CO2. Adherent cells were trypsinized (0.25% Trypsin) for passaging. Both adherent and suspension cells were centrifuged at 550 g for 5 minutes before re-plating in fresh media. HeLa cells, HEK293T cells, and Baby Mouse Kidney (BMK) cells (deficient for *Bax* and *Bak*, kind gift from David Andrews, Sunnybrook) were cultured in DMEM supplemented with 10% FBS and antibiotics (100 mg/mL penicillin and 100 U/mL streptomycin). Jurkat cells were cultured in RPMI supplemented with 10% FBS and antibiotics. SCID.adh cells were cultured in RPMI supplemented with 10% FBS, antibiotics, 1% non-essential amino acids, 1% sodium pyruvate, and 50mM β-mercaptoethanol. OP9-DL4 co-cultures were seeded with LSK (Lin-Sca1+ckit+) cells sorted from bone marrow of WT or ALT-Tg mice, or with GFP+ LSK cells sorted from retrovirally transduced bone marrow, as previously described (35).

### Transfections

Cells were seeded 18 h before transfection, and equal amounts of DNA were transfected into each well using Lipofectamine 3000 Transfection Reagent (Invitrogen; Cat. L3000008) or FuGene HD Reagent (Promega; E2311) in OPTI-MEM. Analysis was performed 24 h post-transfection.

### Electroporation

For immunoprecipitation experiments, Jurkat cells were transfected with 5 μg of vector DNA by Neon Transfection System based on manufacturer’s protocols. After washed with PBS, cells were resuspended in R Buffer to reach a concentration of 2 ×10^7^ cells/ml. Using 100 μl Neon tips, cells were electroporated with the parameter of 1350V, 10 ms, three times, in a Neon tube containing E_2_ Buffer. After the pulse, cells were quickly transferred into a 6-well-plate with 3 ml RPMI supplemented with 10% FBS and antibiotics and cultured for 2 days.

### Dual Luciferase Reporter Assays

Transcriptional activity was assessed by performing Dual Luciferase Reporter (DLR) Assays (Promega; Cat. E1910). The following constructs were co-transfected into cells in 24-well plates using FuGene HD transfection reagent (Promega; Cat. E2311): Renilla Luciferase, 8X-E-box Firefly luciferase reporter construct (pGL3/4 vector) (33), and pCMV-HA HEB expression constructs. Cells were lysed 24 h post-transfection using lysis buffer provided by the DLR assay kit, and luminescence was measured using the BioTek Synergy H1 Hybrid Reader. Firefly Luciferase Units were normalized to Renilla Luciferase Units to calculate Relative Luciferase Units (RLU).

### Retroviral Transduction

GFP (empty vector negative control), HEBAlt (ALT), FFF, EEE, and HEBCan (CAN) retrovirus- producing GP+E cell lines were generated using pMIG-IRES-GFP, pMIG-HEBALT-HA-(WT/FFF/EEE)-IRES-GFP, and pMIG-HEBCAN-HA-IRES-GFP vectors, as previously described (36). BMK cells were co-cultured with the retrovirus-producing cell lines overnight (18h) in media containing Polybrene (10mg/μL). GFP+ cells were sorted by flow cytometry and expanded in culture to create stably expressing cell lines.

### Immunoprecipitation

SCID.adh cells and HeLa cells, or HEK293T cells transfected with expression constructs using Lipofectamine 2000 one day earlier, were harvested using Pierce lysis buffer supplemented with protease (Thermo Scientific, 78425) and phosphatase inhibitors (EMD Millipore, 4906845001). Lysates were incubated on ice for 30 min, with vigorous vortexing every 10 min, and then pre-washed with Pierce Protein A/G agarose slurry for 1 h at 4°C to block non-specific binding. HA-tagged proteins were subjected to IP at 4°C overnight using antibody-agarose beads (Clontech, 631207) followed by three washes with Pierce lysis buffer or were collected anti-HA-conjugated magnetic beads followed by TBST washes (Thermo Scientific, 88836).

### Western Blotting

Cells were lysed using RIPA buffer with protease inhibitors (Halt Protease Inhibitor Cocktail (100X); Thermo Scientific; Cat. 78430). Protein concentrations were measured using the Pierce BCA Protein Assay Kit (Thermo Scientific; 23225) and equal amounts were used in SDS-PAGE analysis. Lysates were denatured in SDS loading buffer (containing DTT), heated at 100°C for 5 min, and loaded onto acrylamide gels. Sizes were determined using the PageRuler Plus Prestained Protein ladder (Thermo Scientific, 10 to 250kDa). Samples were transferred from the gel onto a PVDF membrane (Biorad TransBlot Turbo) by the semi-dry transfer method using the TransBlot Turbo Machine (Biorad), and the membrane was blocked overnight at 4°C on a shaker in 1X TBST (Tris-buffered saline with Tween 20 at 0.1% v/v) with 5% skim milk to eliminate non-specific binding. The next day, the blots were probed with primary antibodies in 1X TBST with 5% BSA, washed, and probed with HRP-conjugated secondary antibodies. After washing in TBST, the blot was visualized using the Clarity ECL kit (Biorad) and the Fusion Fx Chemiluminescence and Fluorescence Imager (Vilber Lourmat). Image quantification was performed using ImageJ software. The primary antibodies used in these studies were the HA-Tag polyclonal antibody (Clontech, 631207), pan anti-HEB antibody (Santa Cruz Biotechnology, A-20, sc-357), anti-GAPDH (mouse, EMD Millipore, MAB374), anti-Tubulin (mouse, SCBT; sc-69970), and anti-Alt (in-house). The secondary antibodies were HRP-conjugated Goat anti-Rabbit (Invitrogen, 626120) and Goat anti-Mouse (BioRad, 1706516). The anti-phosphotyrosine antibody (4G10 Platinum) was directly conjugated to HRP (Sigma-Aldrich 16-316).

### Protein Stability Assays

For the cyclohexamide experiments, cells were treated with 300 μg/mL cyclohexamide (CHX) to block translation elongation 24 h post-transfection. The cells were lysed at 0 h, 0.5 h, 1 h, and 2 h post-CHX-treatment, and lysates were analyzed by Western blotting. Band densities were quantified using ImageJ.

### JAK Inhibition

Jurkat cells were co-transfected with ALT, FFF, EEE or pCMV-HA (negative control) and the 8X E-box luciferase reporter and treated with DMSO (no Ruxolitinib) or 1 mM of the pan-JAK inhibitor Ruxolitinib (Invitrogen, tlrl-rux) for 4 h. Dual luciferase assays were conducted, and the data was depicted as relative luciferase units (RLU) normalized to untreated ALT.

### Mass Spectrometry

HEK293 cells transfected with ALT, CAN, EEE, FFF, TR, or empty vector, or whole thymocytes from littermate ALT-Tg mice with or without Vav-Cre, were lysed using RIPA lysis buffer and subjected to IP using the Pierce™ HA-Tag Magnetic IP/Co-IP Kit (ThermoFisher Scientific; 88838) and anti-HA antibodies. Samples were trypsin-digested and analyzed by mass spectrometry at the SPARC BioCentre facility using a Thermo Scientific Q Exactive HF-X hybrid quadrupole-Orbitrap mass spectrometer (SickKids, Toronto). Scaffold (version Scaffold_5.0.1, Proteome Software Inc., Portland, OR) was used to validate MS/MS based peptide and protein identifications. Peptide identifications were accepted if they could be established at greater than 95.0% probability. Peptide Probabilities from Sequest (XCorr Only) and MS-Amanda Proteome Discoverer were assigned by the Scaffold Local FDR algorithm. Peptide Probabilities from X! Tandem were assigned by the Peptide Prophet algorithm (37) with Scaffold delta-mass correction. Protein probabilities were assigned by the Protein Prophet algorithm (38). A filter of 95% protein identity, 95% peptide identity, and a minimum of 3 spectra were applied to all samples, and then proteins present in the HA negative control were removed.

### Post Translational Modification Site Localization

Scaffold PTM (Proteome Software, Portland, OR) was used to annotate PTM sites derived from MS/MS sequencing results using the site localization algorithm developed by Beausoliel et al. (39). MS/MS spectra identified with modified peptides were identified and AScore values and site localization probabilities were calculated to assess the level of confidence in each PTM localization.

### Generation of Anti-ALT Antibodies

Anti-ALT antibodies were made by Abcam (Cambridge, U.K). A peptide corresponding to the Alt domain coding region was synthesized, conjugated to KHL, and injected into rabbits. After several rounds of boosting, the rabbits were exsanguinated to provide polyclonal stocks. Specificity was confirmed by Western blotting of HEK293T cells transfected with either HEBAlt- or HEBCan- expressing constructs (**Figure S2A**), and Rag2-/- thymocytes, which express abundant HEBAlt protein (**Figure S2B**).

### Statistical Analysis

Data was analyzed using Prism software (GraphPad). Statistical significance was determined by comparing two sets of data with the unpaired two-tailed Student’s t test, where a p value of less than 0.05 fulfilled the criteria of statistical significance. The variation between biological replicates of the same group was depicted by graphing the standard error of the mean as error bars. All data shown are reflective of at least two independent experiments. Results of multiple experiments were pooled, normalized, graphed, and shown as individual data points.

## Results

### Optimal HEBAlt Transcriptional Activity Requires the Last Third of the Alt Domain

We have previously shown that the activity of HEBAlt on an E-box promoter-reporter luciferase construct was lower than HEBCan-induced transcriptional activation (33). This could have been due to the absence of the HEBCan-specific N-terminus, or due to unique properties of the Alt domain. To distinguish between these possibilities, we generated HA-tagged HEBAlt mutant truncation expression constructs using naturally occurring methionines as start codons (**Figures 1C, D**). We removed the first third of the Alt domain (MET1), the first two-thirds, (MET2), the entire Alt domain (TR), or the Alt domain and part of exon 9, which is shared between HEBAlt and HEBCan (MET3) and tested their ability to transactivate the reporter construct as compared with WT (ALT) HEBAlt in HEK293T cells. All constructs were expressed at comparable protein levels (**Figure 1E**). ALT induced luciferase activity was above background levels, in agreement with previous studies (33) (**Figure 1F**). The MET1 and MET2 constructs displayed comparable activity to full length ALT, but the TR and MET3 constructs, which lacked the Alt domain completely, had a ~3-fold decrease in activity. These results indicated that the last third of the Alt domain is required for effective transcriptional activation from a multimerized E box site by HEBAlt.

### The YYY Residues in the Alt Domain Regulate HEBAlt Activity

**Figure 2:**
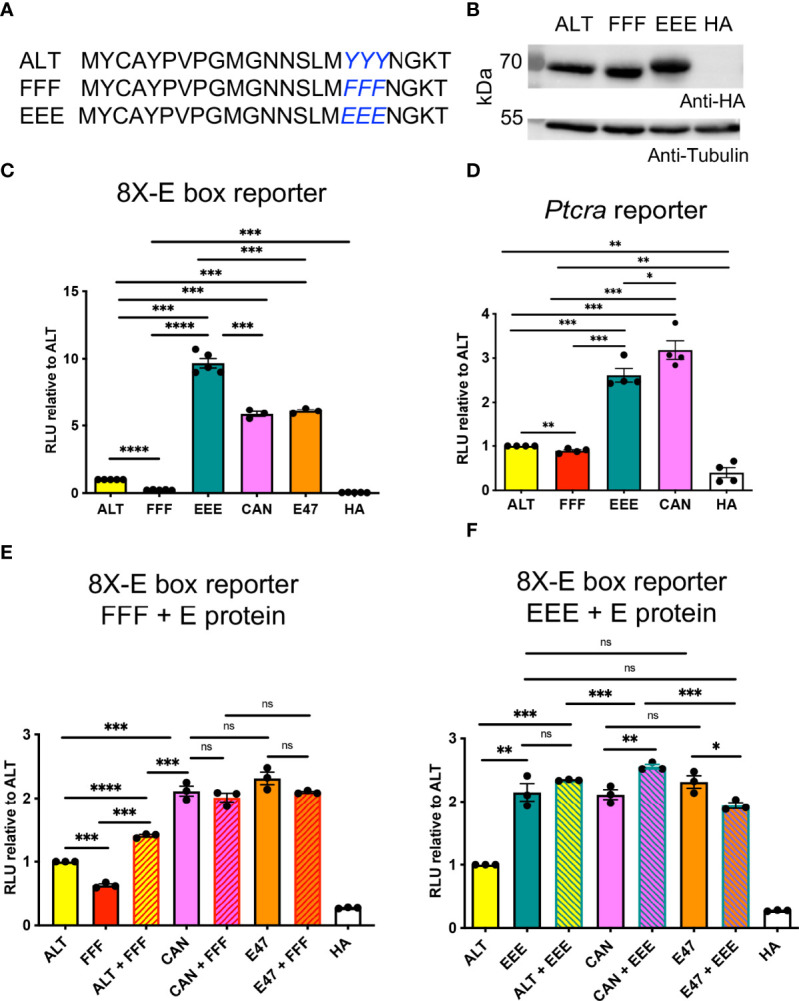
A YYY motif in the Alt domain modulates HEBAlt transcriptional activity. **(A)** Amino acid sequences of the Alt domain of the EEE and FFF constructs. **(B)** Western blot of protein expression for the HA-tagged ALT, FFF, and EEE constructs in HEK293T cells, probed by anti-HA, with anti-tubulin as a loading control. **(C)** Dual luciferase assays on BMK cells transfected with expression constructs for ALT, FFF, EEE, HEBCan (CAN), E2A (E47), or HA empty vector control, along with 8X E box Firefly luciferase and Renilla luciferase constructs. The Y-axis depicts relative luciferase units (RLU) of Firefly to Renilla luciferase values, normalized to ALT. **(D)** Dual luciferase assay on HEK293T cells co-transfected with HA-tagged expression constructs for ALT, FFF, EEE, CAN, or HA empty vector control with a construct in which Firefly luciferase was driven by the Ptcra promoter, and a Renilla luciferase construct. The Y-axis depicts relative luciferase units (RLU) of Firefly to Renilla luciferase values normalized to ALT. **(E)** Constructs were mixed 1:1 to allow the formation of homodimers of ALT, CAN, E2A (E47), or FFF, or heterodimers of ALT, CAN, or E2A with FFF, and activity was measured by luciferase assays using the 8X E box construct. **(F)** Constructs were mixed 1:1 to allow the formation of homodimers of ALT, CAN, E2A (E47), or EEE, or heterodimers of ALT, CAN, or E2A with EEE, and activity was measured in HEK293T cells by luciferase assays using the 8X E box construct. Note that the assays shown in **(E, F)** were conducted at the same time, and the ALT, FFF, EEE, CAN, E47, and HA samples were graphed next to either the FFF or EEE samples for clarity. *P < 0.05, **P < 0.001, ***P < 0.0001, ****P < 0.0001, ns, non-significant.

Closer inspection of the amino acid residues in the Alt domain revealed an abundance of tyrosine (Y) residues, including a triple tyrosine (YYY) motif. To evaluate whether the Alt domain could respond to signaling through tyrosine kinase-mediated pathways, we generated mutant HEBAlt constructs in which the YYY motif was replaced by EEE, imparting a negative charge and mimicking phosphorylation, or FFF, which cannot be phosphorylated (**Figure 2A**). We transfected these constructs into HEK293T cells and confirmed that EEE and FFF were expressed at similar protein levels to ALT (**Figure 2B**). To evaluate the ability of these constructs to activate transcription from the 8X E box reporter construct, we co-transfected them into BMK (Baby mouse kidney) cells. Consistent with our previous findings, luciferase assays showed that ALT had lower levels of activity than HEBCan (CAN) and E2A (E47) (**Figure 2C**). Strikingly, EEE exhibited a ~10-fold increase in activity over ALT, to levels that surpassed CAN. By contrast, FFF activity was ~2-fold lower than ALT. We also tested the activity of these factors in HEK293T cells on the *Ptcra* promoter, which drives expression of the gene encoding pTα in DN thymocytes (40) (**Figure 2D**). Under these conditions, ALT and FFF activity were both low, but EEE approximated HEBCan activity. These results suggest that phosphorylation of the YYY motif in the Alt domain might enable HEBAlt to induce transcription more strongly than HEBCan.

### FFF Does Not Act as a Dominant Negative Form of ALT

To evaluate whether the FFF acted as a dominant negative form of HEB, we assessed whether mixing FFF with ALT, CAN, or E2A would decrease the overall activity when co-transfected into HEK293T cells with the reporter construct. We found that FFF did not interfere with CAN or E2A transcriptional activity but induced a small increase in ALT activity (**Figure 2E**). Next, we asked whether mixing the EEE mutant with each of the wildtype E proteins would impact their activity. No major impact on CAN or E47 activity was observed with the addition or either EEE, suggesting that a negative charge on one E protein within an E protein dimer is sufficient to drive enhanced transcriptional activation (**Figure 2F**).

### HEBAlt Activity Is Reduced by JAK Inhibitors

**Figure 3:**
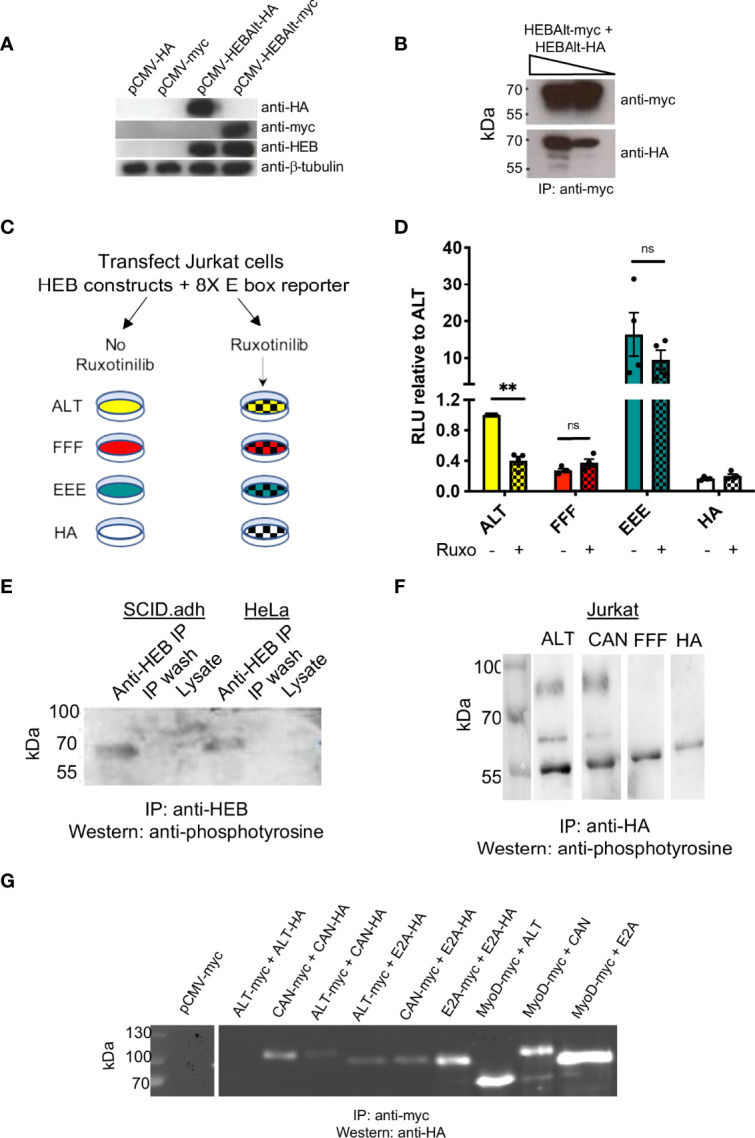
HEBAlt transcriptional activity is decreased by JAK inhibition. **(A)** Western blot of lysates from HEK293T cells transfected with HEBAlt-HA, HA empty vector (pCMV-HA), HEBAlt-myc, or myc empty vector (pCMV-myc) expression constructs probed with anti-HA, anti-myc, anti-HEB (A20) or β-tubulin as a loading control. **(B)** Western blots of HEK293T lysates from co-IPs of HEBAlt-HA and HEBAlt-myc, using anti-myc antibodies for co-IP and anti-HA antibodies for Western protein detection, at a higher (left) or lower (right) concentration of lysate (triangle). **(C)** Diagram of JAK inhibition experimental design. Jurkat cells were co-transfected with HEBAlt (ALT; yellow), FFF (red), EEE (green) or HA empty vector (white), and 8X E box Firefly luciferase and Renilla luciferase constructs. Cells were cultured with (checked) or without (filled) the pan-JAK inhibitor Ruxotinilib (Ruxo) for two days at 1 mM, followed by dual luciferase assay. **(D)** Activation of the reporter construct as measured by dual luciferase assay. The Y-axis depicts relative luciferase units (RLU) of Firefly to Renilla luciferase values normalized to ALT. **P < 0.001, ns, non-significant. **(E)** Detection of endogenous tyrosine phosphorylated protein in the mouse pro-T cell line SCID.adh and human HeLa cells after immunoprecipitation with anti-HEB antibodies, assessed by probing of Western blot with anti-phosphotyrosine antibodies. **(F)** Western blot of lysates extracted from Jurkat cells transfected with ALT or CAN, IP’d with anti-HA, and probed with anti-phosphotyrosine antibodies. **(G)** Expression proteins epitope tagged with myc or HA were contransfected into HEK293T cells and co-immunoprecipitated with anti-myc. Lysates were analyzed by Western blots probed with anti-HA. The discrepancy between ALT-ALT dimerization seen in **(B)** versus **(G)** is likely due to the exposure times on the different blots, since a far longer detection time was necessary to observe ALT-ALT dimers, and the blot in **(G)** was optimized to visualize the range of intensity for the other dimers.

To formally assess whether ALT proteins can form homodimers, we generated a myc-tagged ALT construct (**Figure 3A**). ALT-HA and ALT-myc were co-transfected into HEK293T cells, and antibodies were used to immunoprecipitate (IP) ALT-myc. Western blot analysis of HA-tagged constructs clearly showed that ALT-myc and ALT-HA can form a complex (**Figure 3B**), consistent with the existence of HEBAlt homodimers. Next, we assessed whether JAK tyrosine kinases could enhance HEBAlt activity. ALT or mutant constructs and 8X-E box reporter constructs were co-transfected into Jurkat T cells, which provide a background of low constitutive JAK signaling (41). ALT, FFF, EEE, or control empty vector (HA) transfected Jurkat cells were cultured with or without the JAK inhibitor Ruxolitinib for two days, followed by luciferase assays (**Figure 3C**). As before, FFF displayed lower activity than ALT. In contrast, EEE exhibited a ~20-fold higher activity than ALT (**Figure 3D**). Importantly, JAK inhibition decreased ALT activity but did not significantly affect the activity of FFF or EEE. These results indicate that modulation of JAK function can regulate HEBAlt activity in a YYY-dependent manner.

### Phosphorylation of the YYY Motif in the Alt Domain

To assess whether HEBAlt could be phosphorylated, we used anti-HEB (A20) to IP endogenous HEB factors from HeLa and SCID.adh cells (42) (**Figure 3E**). Anti-HEB was used to IP HEB factors, and the Western blot was probed using anti-phosphotyrosine antibodies (4G10). One band around 60 kDa was detected, consistent with phosphorylation of HEBAlt tyrosine residues. To verify the specificity of the endogenous bands, we transfected HA-tagged ALT, CAN, or FFF constructs into Jurkat cells and cultured them for two days. The transfected Jurkat cells were subjected to IP with anti-HA antibodies (**Figure 3F**) and Western blots were probed with anti-phosphotyrosine antibodies. In the ALT and CAN transfected Jurkat cells, we observed two bands, one at the size of HEBCan (upper band; ~100 kD) and another at the size of HEBAlt (lower band; ~60 kD), whereas the FFF sample lacked bands indicating phosphorylated tyrosine. To assess whether the presence of two bands in the each of the ALT and CAN samples might be due to enrichment of complexes containing both HEBAlt and HEBCan, we performed co-IPs in HEK293T cells with myc-tagged and HA-tagged HEBAlt, HEBCan, E2A, and MyoD (**Figure 3G**). MyoD is a Class II bHLH factor that binds more strongly to E proteins than they do with each other. Our results showed that HEBAlt can form heterodimers with HEBCan, E2A, and MyoD. Therefore, the presence of a phosphorylated CAN band in the ALT sample and a phosphorylated ALT band in the CAN sample suggests that both proteins were precipitated as components of complexes containing HA-tagged proteins. Moreover, these bands suggest that both HEBCan and HEBAlt, whether exogenous or endogenous, can be tyrosine phosphorylated, whereas FFF cannot. Moreover, the lack of bands in the FFF lane suggest that it may not be able to form stable dimers with either HEBAlt or HEBCan, providing a partial explanation for its decreased function.

### Detection of a Phosphorylated YYY Motif Within the E2-2 Alt Domain

**Figure 4:**
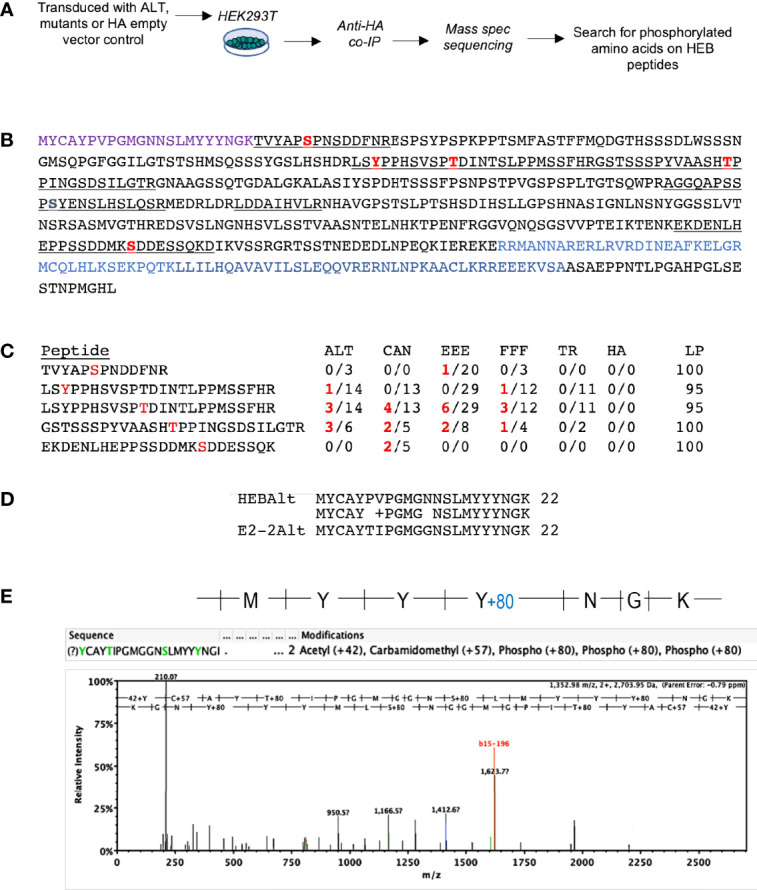
Detection of post-translational modifications in HEBCan, HEBAlt, and HEBAlt mutant proteins transfected into HEK293T cells. **(A)** Diagram of experimental design. HEK293T cells were transfected with HA-tagged constructs and subjected to immunoprecipitation 24 hrs later using anti-HA antibodies. Lysates were subjected to mass spectrometry sequencing, and sequences were analyzed to detect post-translational modifications (PMTs). **(B)** Annotated amino acid sequence of HEBAlt. Purple = Alt domain. Underlined = peptides detected. Red = residues with S/T/Y phosphorylation detected at least once. Blue = bHLH domain. No peptides were detected representing the Alt domain. **(C)** Table showing the numbers and distribution of specific PMTs in each peptide among samples bearing different constructs. LP = probability that the site of phosphorylation has been correctly annotated, as calculated by the Scaffold-PTM software. **(D)** Alignment of the Alt domains of HEB and its paralog E2-2 showing the two amino acid difference between them. **(E)** Spectrum of a peptide identified as E2-2Alt in the FFF sample with a mass shift of +80 at the last tyrosine of the YYY motif, consistent with phosphorylation.

To directly identify phosphorylated residues on HEBAlt, we transfected HEK293T cells with HA-tagged ALT, FFF, EEE, CAN, and TR. After 24 h, protein lysates were generated in the presence of phosphatase inhibitors and subjected to IP with anti-HA. The precipitated proteins were subjected to mass spectrometry sequencing, and ScaffoldPTM was used to identify phosphorylated residues on ALT, FFF, EEE, CAN, and TR (**Figure 4A**). Five unique peptides were recovered from this set of samples, all of which were located downstream of the ALT/CAN junction (**Figures 4B, C**). Spectra consistent with phosphorylation were detected on two serines, two threonines, and one tyrosine. These were sparsely present among the peptides and were differentially represented in each sample (**Figure 4C**). However, we also detected a paralog of HEBAlt, E2-2Alt (43), likely due to co-immunoprecipitation with HEBAlt. The Alt domains of E2-2 and HEB differ by two amino acids (**Figure 4D**), allowing unequivocal identification. This spectrum showed a +80 shift on the last tyrosine of the YYY motif indicating the presence of a phosphate group (**Figure 4E**). It should be noted that E2-2Alt spectra represented an endogenous protein that is not overexpressed, further supporting the capacity of the YYY motif in E protein ALT variants to be directly phosphorylated on at least one tyrosine residue.

### A Transgenic Mouse Model for Studying HEBAlt *In Vivo*

**Figure 5:**
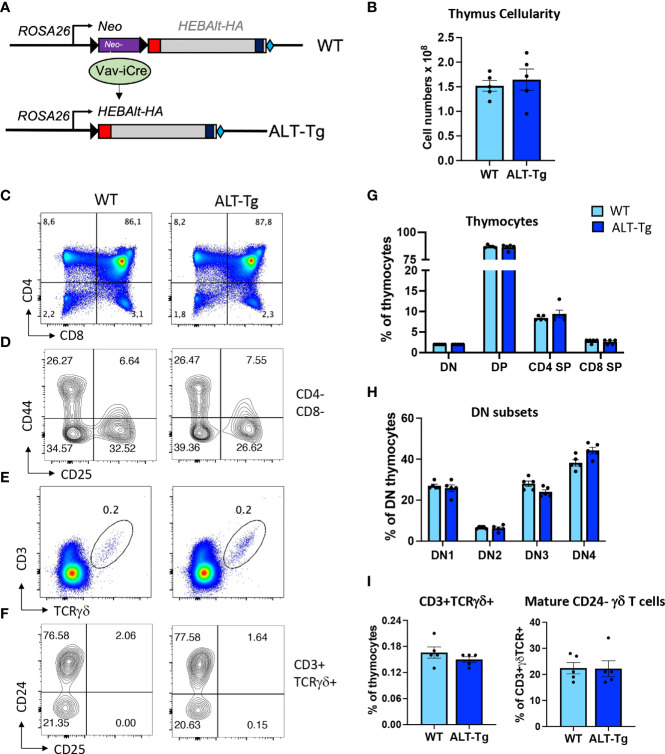
Generation and characterization of conditional HEBAlt-HA transgenic (ALT-Tg)_ mice. **(A)**. An HA-tagged HEBAlt coding cassette was inserted into the ROSA26 locus downstream of a loxP-Neo-stop-loxP cassette in ES cells, giving rise to mice carrying an inducible HEBAlt-HA transgene. These mice were bred to Vav-iCre mice to generate WT (Vav-Cre-) and ALT-Tg (Vav-Cre+) littermates. **(B–E)**. Phenotype of thymic developmental subsets in WT and ALT-Tg mice by flow cytometry within total thymocytes **(B, D)** and and within DN cells **(C, E)**. **(F–H)**. Percentages of γδ T cells within total thymocytes **(F, H)**, and percentages of immature (CD24+) and mature (CD24-) cells within the CD3+TCRγδ+ populations **(G, H)**.

Given the lack of reagents available for studying HEBAlt, we generated several new tools to move our studies into the context of T cell development. The first was an antibody that detects the Alt domain (**Figure S2**). These antibodies (anti-ALT) worked well for detection of HEBAlt using Western blots but not for immunoprecipitations. Therefore, we designed a new mouse model with inducible expression of HEBAlt. To enhance the versatility of these mice, we inserted an HA-tagged HEBAlt construct into a loxP-stop-loxP cassette driven by the Rosa26 constitutively expressed promoter to generate ALT-Tg mice (**Figure 5A**). In these mice, the HEBAlt-HA transgene is silent until the stop cassette is removed by Cre-mediated excision, enabling induction of HEBAlt-HA expression in a stage- and lineage-specific manner. We bred these mice to Vav-iCre mice to induce deletion in all hematopoietic cells, and generated ALT-Tg^Vav-iCre^ (ALT-Tg) mice, which were compared with ALT-Tg (WT) littermates containing the transgene in the absence of Cre.

### Phenotyping of Major Thymocyte Subsets in ALT-Tg Mice at Steady State

**Figure 6:**
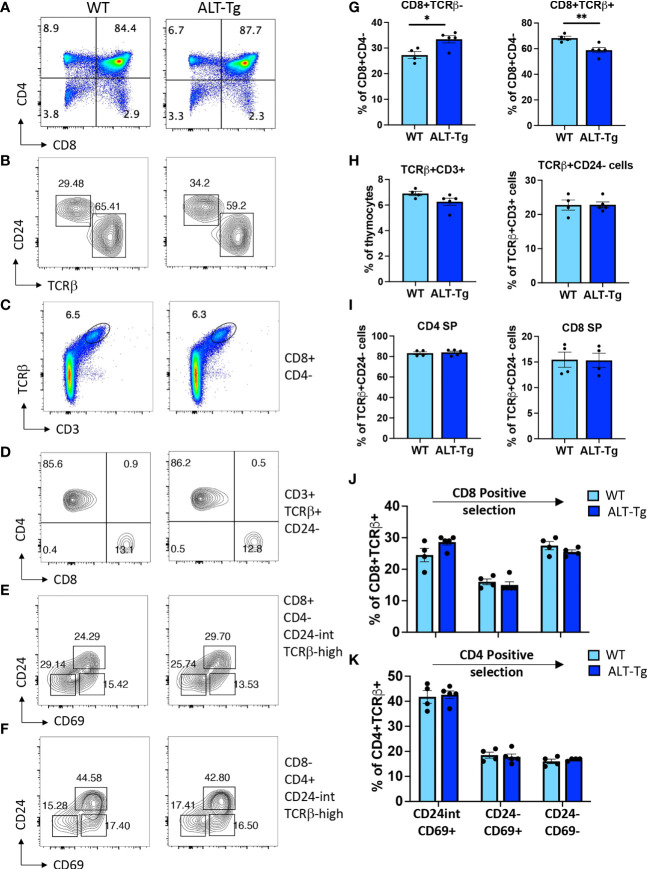
Perturbation of the ISP to CD8 SP thymocyte ratio in ALT-Tg mice. **(A)** Flow cytometry plot showing the gates for CD4+ CD8- and CD4- CD8+ cells. **(B)** Flow cytometry plots showing immature (CD24-hi TCRβ-) and mature (CD24-low TCRβ+) subsets in the CD8+ subset gated as in **(A)**. **(C)** Flow cytometry plots showing the percentages of TCRβ+ CD3+ cells within the total thymocyte population. **(D)** Flow cytometry plot of the percentages of CD4 and CD8 cells within the TCRβ+ CD3+ CD24- population. **(E)** Flow cytometry plot of CD8+ TCRβ+ CD24- cells passing through each stage of positive selection as assessed by sequential downregulation of CD24 and CD69. **(F)** Flow cytometry plot of CD4+ TCRβ+ CD24- cells passing through each stage of positive selection as assessed by sequential downregulation of CD24 and CD69. **(G)** Quantification for replicates of the plots shown in **(B)**. **(H)** Quantification for replicates of the plots shown in **(C)** and for the percentages of CD24- cells within the TCRβ+ CD3+ population, which are not shown as FACS plots. **(I)** Quantification for replicates of the plots shown in **(D)**. **(J)** Quantification for replicates of the plots shown in **(E)**. **(K)** Quantification for replicates of the plots shown in **(F)**. *P < 0.05, **P < 0.001.

The ALT-Tg mice had no obvious defects at the level of gross morphology. The adult thymus had normal cellularity (**Figure 5B**) and undisturbed proportions of DN, DP, and SP thymocytes at steady state (**Figures 5C, G**), as well as a normal distribution of DN subsets (**Figures 5D, H**). We also evaluated the percentages of γδ T cells (**Figures 5E, I**), and the distribution of immature versus mature cells within the γδ T cell subset according to expression of CD24 (**Figures 5F, I**). No significant differences were observed. We next undertook a more in-depth analysis of thymocyte subsets that normally do not express HEBAlt at the mRNA level (**Figure 6**). Gating on the CD8+CD4- subset (**Figure 6A**) revealed that the proportion of immature (CD24+ TCRβ-) cells to mature (CD24-low TCRβ+) cells was higher in ALT-Tg mice than in WT mice, with a corresponding decrease in the percentage of mature CD8+ SPs in the ALT-Tg mice (**Figures 6B, G**). This was not accompanied by a decrease in the percentage of DP cells, suggesting the increase in CD8+ ISP did not indicate a developmental block at the DN to DP transition. To assess the ratio of mature CD4 to CD8 cells, we first gated on TCRβ+ CD3+ cells (**Figures 6C, H**), and then on CD24- cells within that population (**Figure 6H**). These two populations were present at similar frequencies in WT and ALT-Tg. We next evaluated the frequencies of CD4 and CD8 cells within the mature (TCRβ+ CD3+ CD24-) population and found no differences between WT and ALT-Tg mice (**Figures 6D, I**). To assess whether positive selection was impaired in ALT-Tg mice, we examined CD24 and CD69 expression within the TCRβ+ CD8+ (**Figures 6E, J**) and the TCRβ+ CD4+ (**Figures 6F, K**) populations. Thymocytes from ALT-Tg mice exhibited transient upregulation of CD69 accompanied by CD24 downregulation comparable to WT thymocytes in both the CD4+ and CD8+ SP subsets. Therefore, we found no evidence for alterations in positive selection in the ALT-Tg mice on the C57Bl/6 background with a polyclonal repertoire.

### HEBAlt Protein Persists in Thymocytes After mRNA Expression Ceases

**Figure 7:**
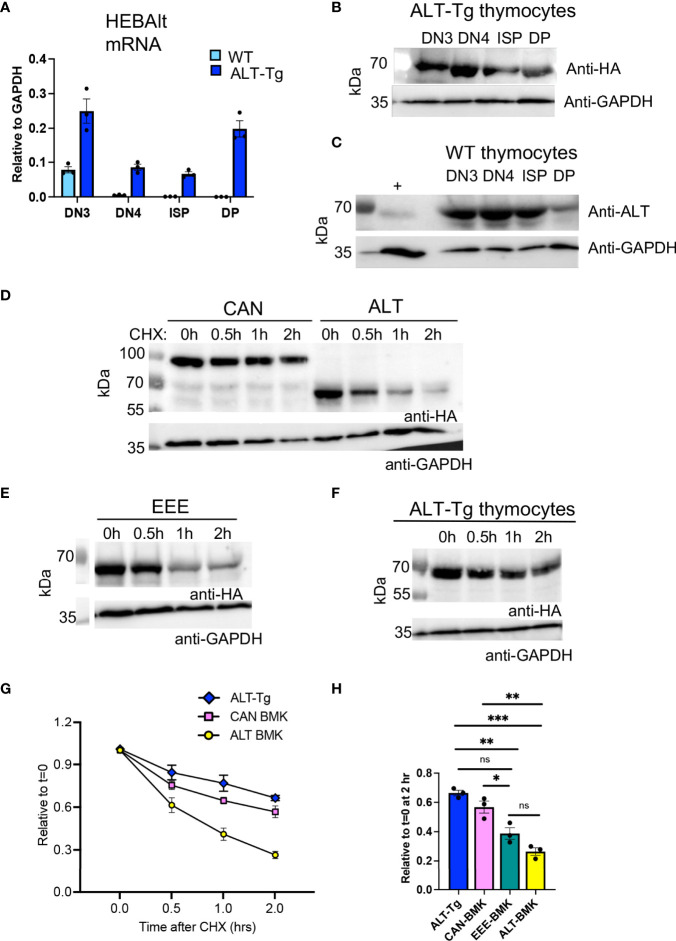
Differential protein stability of HEBAlt in T cell and non-T cell contexts. **(A)** Levels of HEBAlt mRNA in sorted thymocyte subsets from WT and ALT-Tg mice as assessed by qRT-PCR. **(B)** Levels of HEBAlt-HA transgene-derived protein in sorted thymocyte subsets from ALT-Tg mice as determined by Western blots probed with anti-HA or anti-GAPDH as a loading control. **(C)** Levels of endogenous HEBAlt in sorted thymocyte subsets from WT mice as determined by Western blot probed with anti-ALT specific antibodies or anti-GAPDH as a loading control. **(D–F)**. BMK cells were transduced with constructs expressing HA-tagged **(D)** HEBCan (CAN), HEBAlt (ALT) or **(E)** EEE expression vectors and examined for protein stability in parallel with ALT-Tg thymocytes **(F)**. Cells were treated with DMSO or 300 mg/mL cycloheximide (CHX) for 0, 0.5, 1, or 2 h. C, and protein was measured by Western blot detecting using anti-HA or anti-GAPDH as a loading control. **(G)** Time course of quantified band densities (n = 3 independent experiments), relative to GAPDH at t=0 and normalized to untreated sample (t = 0). EEE is not shown to enhance clarity of the plot. **(H)** Comparison of all samples at t = 2 hr of CHX treatment, including EEE. *P < 0.05, **P < 0.001, ***P < 0.0001, ns, non-significant.

To confirm expression of the HEBAlt transgene in ALT-Tg mice, we measured HEBAlt mRNA and protein levels (**Figure 7**). Although HEBAlt mRNA decreased at the DN3 to DN4 transition in the WT mice, as expected, it was sustained up to the DP stage in ALT-Tg thymocytes, as detected by anti-HA (**Figure 7A**). Western blots of sorted thymocyte subsets showed that the transgenic HEBAlt protein was also expressed up to the DP stage, as detected by anti-HA (**Figure 7B**). Surprisingly, we also found that endogenous HEBAlt protein, as detected by the anti-ALT antibody, was present in WT thymocytes subsets that had downregulated HEBAlt mRNA (**Figure 7C**). These observations suggest that HEBAlt protein stability may be enhanced across the β-selection checkpoint.

### The Thymocyte Environment Enhances HEBAlt Protein Stability

Given the discordance between HEBAlt mRNA and HEBAlt protein expression, we evaluated HEBAlt protein stability by performing cycloheximide chase experiments. BMK cells were stably transduced with HA-tagged HEBAlt or HEBCan to provide a non-T cell context for protein stability studies, and these were compared with HA-tagged ALT-Tg thymocytes. Cycloheximide was added to the cells to stop *de novo* protein synthesis, and then washed out (chase). Samples were taken at 0, 0.5, 1, and 2 h after chase, and protein levels were evaluated using Western blots probed with anti-HA and anti-GAPDH. In BMK cells, HEBAlt and HEBCan were expressed at comparable amounts at t=0, but HEBAlt protein levels dropped more precipitously than HEBCan levels, indicative of decreased stability (**Figure 7D**). By contrast, HEBAlt protein appeared more stable in thymocytes than in BMK cells (**Figures 7F, G**), and the levels were statistically indistinguishable from HEBCan in BMK cells after 2 hrs (**Figure 7H**). EEE showed only a minor improvement in HEBAlt stability in BMK cells (**Figures 7E, H**), suggesting that factors other than YYY phosphorylation were at least partially responsible for this feature of HEBAlt biology.

### Transgenic HEBAlt Protein is Reduced in Non-Thymic Hematopoietic Cells

**Figure 8:**
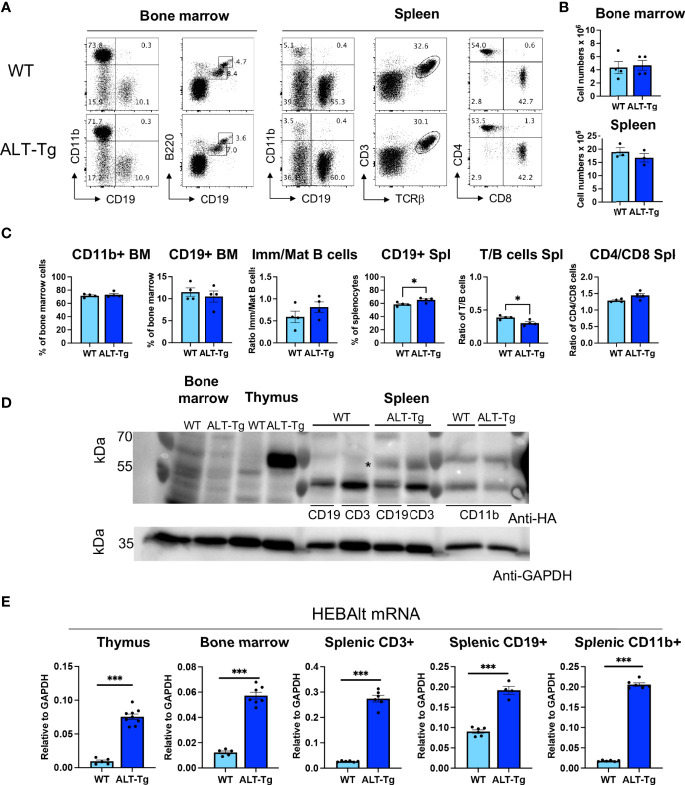
Loss of HEBAlt protein stability in immune cell subsets outside the thymus. A-C. **(A)** Phenotype, **(B)** cell numbers, and **(C)** frequencies or ratios of T cell, B cell, and myeloid subsets in bone marrow and spleen of WT (light blue) and ALT-Tg (dark blue) mice. The bone marrow B220/CD19 plot is gated on CD19+ cells, and the spleen CD4/CD8 plot is gated on CD3+ cells. **(D)** Western blot analysis of transgene-derived HEBAlt-HA protein as detected by anti-HA in WT or ALT-Tg bone marrow, thymocytes, or sorted splenic T (CD3), B (CD19), and myeloid (CD11b) cells. GAPDH is shown as a loading control. The star pinpoints a band that is present in the ALT-Tg splenic T and B cells but not in the WT cells or myeloid cells. **(E)** Expression of total HEBAlt levels in thymus, bone marrow, and splenic T, B, and myeloid cells from WT and ALT-Tg mice, as analyzed by qRT-PCR and normalized to GAPDH, showing that there are higher levels of HEBAlt mRNA in these populations than in WT mice, despite the paucity of transgene-derived HEBAlt protein. *P < 0.05, ***P < 0.0001.

We have shown in previous studies that forced expression of HEBAlt was inhibitory to the development of non-T cell lineages *in vitro* (33). We therefore analyzed the proportions of myeloid cells (CD11b^+^), B cells (CD19^+^), and the ratio of immature (B220^int^ CD19 ^int^) to mature B cells (B220^hi^ CD19^hi^) in the bone marrow (**Figure 8A**). We found no significant differences between the WT and ALT-Tg mice. Absolute numbers of bone marrow cells and splenocytes were also indistinguishable (**Figure 8B**). We also analyzed the percentages of B cells, the ratios of T to B cells, and the ratios of CD4 to CD8 T cells within the CD3^+^TCRβ^+^ population in the spleen. There was a slight decrease in the ratio of T cells to B cells in the ALT-Tg spleen, but otherwise no major perturbations were observed (**Figures 8A, C**). Given our observation of differential HEBAlt protein stability in transduced BMK cells versus ALT-Tg thymocytes, we next analyzed the expression of the HEBAlt transgene in spleen and bone marrow subsets from ALT-Tg mice. Thymocytes, RBC-depleted bone marrow cells and splenocytes that had been sorted to obtain T (CD3+), B (CD19+), and myeloid CD11b+ (myeloid)-enriched samples, and these samples were subjected to Western blot analysis with anti-HA. Transgenic HA-tagged HEBAlt protein was expressed strongly in ALT-Tg thymocytes but was undetectable in ALT-Tg bone marrow (**Figure 8D**). Splenic B and T cells had similar levels of HEBAlt-HA protein expression, whereas none was apparent in the myeloid fraction. However, qRT-PCR showed that ALT-Tg cells had higher overall levels of HEBAlt mRNA than their WT littermates in all three tissues, confirming expression of the transgene at the RNA level (**Figure 8E**). Interestingly, HEBAlt-HA protein levels were much stronger in thymocytes than they were in the splenic T or B cells. These results suggest that the thymus provides a protective environment for HEBAlt stability, that this protection fades in mature T cells, and that it is absent in myeloid cells.

### ALT-Tg Thymocytes Exhibit a Delay in T Cell Development *In Vitro*

**Figure 9:**
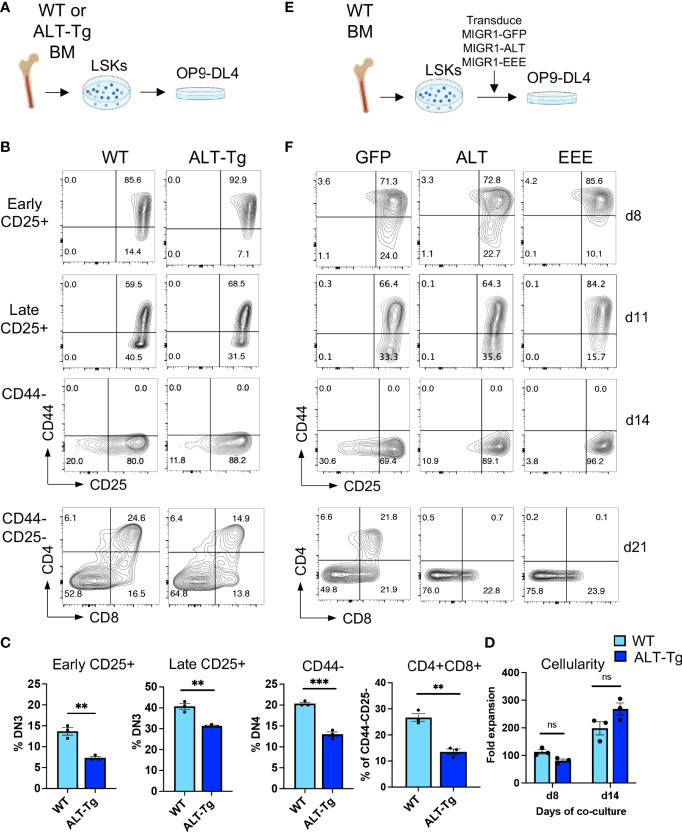
Unrestrained activity of HEBAlt inhibits progress through T cell development. **(A)** Diagram of experimental procedure using precursors from ALT-Tg mice. **(B)** Flow cytometry analysis of progression through T cell development by bone marrow-derived LSKs from ALT-Tg mice co-cultured on OP9-DL4 cells. Gates are shown to the left of each plot. **(C)** Quantification of the populations defined by flow cytometry in **(C)**. **(D)** Fold expansion of cells after 8 or 14 days of OP9-DL4 co-culture. **(E)** Diagram of experimental procedure using precursors from WT mice transduced with retroviral vectors encoding GFP (vector only), ALT, or EEE. **(F)** Flow cytometry analysis of T cell development. Gates are the same as shown in **(B)**. **P < 0.001, ***P < 0.0001, ns, not significant.

Considering these observations, we reasoned that the dynamics of steady state T cell development might mask changes caused by elevated levels of HEBAlt in thymocytes in the ALT-Tg mice. We therefore sorted bone marrow Lin-Sca1+Kit+ (LSK) progenitors from WT and ALT-Tg mice and placed them in OP9-DL4 co-cultures (**Figure 9A**), which allowed us follow T cell differentiation over time (**Figures 9B, C**). We found that ALT-Tg cells were subject to developmental delays compared to their WT counterparts, both at early and at later timepoints (**Figure 9C**). No major changes in cellularity were noted (**Figure 9D**), suggesting that these changes were not due to defects in cell growth or survival. Instead, these results indicate that disrupting the normal balance of HEBAlt in T cell precursors partially inhibits T cell development.

### Forced Expression of EEE Inhibits T Cell Development at Multiple Stages

We next evaluated the impact of forced expression of the EEE mutant on T cell development. WT bone marrow precursors were transduced with MIGR1-based retroviral vectors encoding HEBAlt (ALT), EEE, or empty vector. GFP+ LSK cells were sorted and placed in OP9-DL4 cell co-cultures for varying amounts of time (**Figure 9E**). Interestingly, progression of ALT-transduced cells from the DN2 to DN3 stages was similar to that seen in WT cultures, whereas the EEE cells exhibited a strong block at this transition (**Figure 9F**). However, both ALT and EEE cultures exhibited a partial block in the DN3 to DN4 transition and failed to progress to the DP stage. Therefore, dysregulation of HEBAlt expression and/or activity can interfere with T cell development.

### HEBAlt Is Present in Complexes Containing STAT1 in ALT-Tg Thymocytes

**Figure 10:**
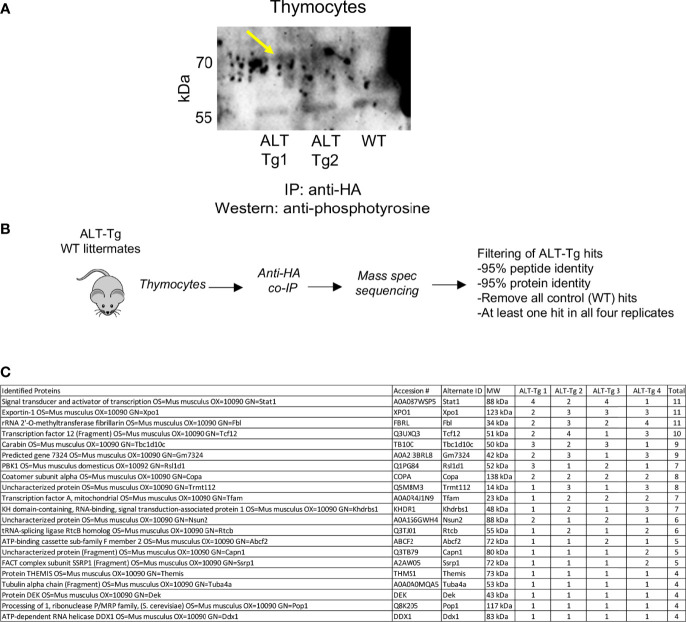
Identification of potential proteins in HEB-containing complexes in ALT-Tg thymocytes. **(A)** Western blot of thymocyte lysates from two ALT-tg mice and one WT littermate immunoprecipitated with anti-HA and probed with anti-phosphotyrosine. The yellow arrow indicates the bands of ~70 kD, corresponding to the size of HEBAlt, that are present in both ALT-Tg samples but not in the WT sample. **(B)** Diagram of experimental design and protocol. **(C)** Identities, molecular weights, and numbers of spectra detected across four replicates for all proteins identified as potential HEBAlt complex partners by the criteria shown in **(B)**.

We next set out to determine whether the HEBAlt-HA protein was tyrosine phosphorylated in ALT-Tg thymocytes (**Figure 10**). Unfractionated thymocytes from ALT-Tg or WT littermates were isolated and subjected to co-IP with anti-HA antibodies, followed by Western blot analysis of tyrosine phosphorylation (**Figure 10A**). These results clearly showed that transgene-derived HEBAlt can be tyrosine phosphorylated in ex vivo thymocytes from ALT-Tg mice. To identify putative protein partners of HEBAlt in this context, we subjected WT and ALT-Tg thymocytes to IP using anti-HA antibodies. Two independent experiments were performed, each of which contained biological duplicates (**Figure 10B**). The IP fractions were subjected to trypsin-mediated digestion, followed by mass spectrometry sequencing. The data was analyzed using Scaffold5, with a cut-off of 95% protein identity and 95% peptide identity. Spectra represented in the littermate controls were used to screen out background hits. Twenty-two proteins represented by at least one spectrum all four replicates were identified (**Figure 10C**). HEBAlt was represented in all four samples for a total of 10 spectra but was not found at all in the negative control samples, consistent with IP-mediated enrichment of HEBAlt-HA. The two most enriched proteins were STAT1, a transcription factor that is phosphorylated and activated by JAK, and Xpo1, which is involved in nuclear export. These results suggest that HEBAlt, like STAT1, can be shuttled between the nucleus and the cytoplasm by Xpo1 (44) in response to cytokine signaling, and that HEBAlt and STAT1 may be coordinately regulated.

## Discussion

E proteins are essential regulators of T cell development, but the relative roles of HEB and E2A are not well understood. Here, our studies revealed that HEBAlt functions as a hub for integrating cytokine signaling and E protein activity in a restricted cellular context. This restriction occurs at multiple levels, including mRNA expression, protein stability, and post-translational modifications. Breeching these controls by extending HEBAlt expression past the point at which it is normally turned off, or by bypassing the need for signal-dependent phosphorylation, impeded T cell development, exposing a need for strict control of HEBAlt function. Moreover, our results revealed that the unique YYY motif in the Alt domain provides a mechanism for sensing changes in extracellular cues and may provide an axis for the coordinated regulation of E proteins with other JAK-responsive factors, such as STATs. Thus, the downregulation of HEBAlt at the DN to DP transition may be necessary to remove a mode of E protein activity that is beneficial to early T cell precursors but harmful to later stages of thymocyte development.

HEB proteins are essential to inhibit the development of ILCs even in the presence of Notch ligands (10, 45). Since ILCs require IL-7R signaling, it may be that elevating HEBAlt activity would aid in restricting IL-7R expressing precursors from adopting an ILC fate. This would be especially important at the DN2 stage, when most cells express cell surface IL-7R. By contrast, DN3 cells are more heterogeneous with respect to levels of IL-7R expression (46). In the adult thymus, a gradient of IL-7 exists that is highest near the corticomedullary junction and lowest near the subcapsular zone (SCZ), where β-selection takes place (47, 48). Therefore, decreasing HEBAlt activity may function to allow β-selection once DN3 cells as they move into the outer cortex where concentrations of IL-7 are low. This would be consistent with the observation that HEB protein is degraded in thymocytes undergoing leukemic transformation, resulting in a decrease in Cdkn1a expression and dysregulated proliferation, in both mice and humans (49).

A connection between E proteins, JAK, and Suppressor of Cytokine Signaling (SOCS) has been previously proposed, in which JAK activate STATs to provide a positive input into E2A activity (50). E2A then upregulates SOCS to inhibit cytokine receptor signaling. We propose that HEBAlt provides the missing link between JAK and E protein activity in early T cell precursors. It remains to be seen whether HEBAlt is directly involved in the regulation of SOCS function. Loss of IL-7R signaling may also be required to silence expression of TCRγ regulatory elements that are activated by STATs (51, 52). Therefore, residual HEBAlt function after b-selection may help to enforce the αβ T cell fate.

Our complimentary approaches using co-cultures and mouse models allowed us to analyze a single wave of T cell development *in vitro* and to monitor T cell development at steady state *in vivo*. Progenitors from ALT-Tg mice in OP9-DL4 co-cultures showed a much stronger defect in T cell development than we observed *in vivo*, akin to what has been previously observed for HEB-deficient and TCF1-deficient mice (21, 53). Given our findings, this may be due in part to the movement of progenitors through different niches in the intact thymus, resulting in modulation of IL-7 availability and regulation of signaling environments that contribute to HEB protein stability (49). In ALT-Tg mice, the mild differences observed in the ISP and CD8 SP subsets were not reflected in the subsets representing progress through positive selection in a background with a polyclonal TCR repertoire. Additional studies of ALT-Tg on backgrounds with fixed TCR transgenes would be helpful in further examining the impact of inappropriate HEBAlt expression on thymocyte maturation.

The modulation of HEBAlt protein stability in different cellular contexts was remarkable. First, we observed that HEBAlt protein persists across the β-selection checkpoint into the ISP stage after its mRNA expression was extinguished. Many studies have described signal-dependent inhibition of E protein activity or stability. These signaling pathways include AKT, casein kinase II, Notch1, MAPK, and calmodulin (54–56, 57). However, it has also been shown that TCF1 protects HEB from degradation in DP thymocytes (58). Therefore, interaction with TCF1 may be one mechanism by which HEBAlt protein could be stabilized after its mRNA expression ceases. This would be consistent with our observation that HEBAlt expressed in the thymocytes of ALT-Tg mice are more stable than those in BMK cells.

Secondly, our results showed that HEBAlt protein was undetectable in non-lymphoid cells in the bone marrow and spleens of ALT-Tg mice, despite strong expression of HEBAlt mRNA. This could be due to fact that myeloid cells express high Id2 levels. Id2 undergoes proteasome-mediated degradation (59), and therefore could destabilize associated E proteins. Thus, our transgenic approach revealed another layer of regulation that could inhibit the induction of E protein target genes outside the lymphoid context. We also observed that thymocytes from ALT-Tg mice had much stronger expression of HEBAlt protein than peripheral T and B cells. Therefore, it is likely that a combination of positive and negative inputs regulates HEB stability in different cellular contexts. Moreover, our results indicate that HEBAlt can provide a unique input to the signals that converge on E protein function to control protein stability and activity.

Taken together, we have defined a new mode of E protein control that links cytokine signaling to E protein activity, and which may be essential for the gatekeeping function exhibited by E2A and HEB prior to β-selection. Additional studies will be needed to determine whether YYY phosphorylated HEBAlt binds to the same gatekeeping target genes as HEBCan and E2A, or whether it imposes another type of control by interaction with distinct protein partners downstream of IL-7R such as STATs or PI3K-responsive transcription factors.

## Data Availability Statement

The original contributions presented in the study are included in the article/**Supplementary Materials**. The mass spectrometry datasets generated for this study can be found in the MassIVE Repository, identifier MSV000088631 (HEK293T cells transfected with HA-tagged HEB constructs) and MSV000089563 (HEBAlt-transgenic thymocytes). Further inquiries can be directed to the corresponding author.

## Ethics Statement

The animal study was reviewed and approved by Sunnybrook Research Institute Animal Care Committee.

## Author Contributions

KY, MA, and JZ-P conceived of and designed the study. KY, AY, JR, AT, LW, and MM generated the data. KY, AT, AY and MA analyzed the data and wrote the manuscript. MA and JZ-P provided funding for the study. All authors contributed to the article and approved the submitted version.

## Funding

This work was supported by grants from the NIH (1P01AI102853-06) to JZ-P and MA, and from CIHR (201610PJT) and NSERC (RGPIN 05333-14) to MA. KY was supported by an NSERC fellowship.

## Conflict of Interest

The authors declare that the research was conducted in the absence of any commercial or financial relationships that could be construed as a potential conflict of interest.

## Publisher’s Note

All claims expressed in this article are solely those of the authors and do not necessarily represent those of their affiliated organizations, or those of the publisher, the editors and the reviewers. Any product that may be evaluated in this article, or claim that may be made by its manufacturer, is not guaranteed or endorsed by the publisher.
