# Supplementary material for: Regulation of the Signal-Dependent E Protein HEBAlt Through a YYY Motif Is Required for Progression Through T Cell Development
Source: Front Immunol. 2022 Aug 3;13:848577. doi: 10.3389/fimmu.2022.848577 (PMC9385190; doi:10.3389/fimmu.2022.848577)
Supplement: Supplementary file 1 [file DataSheet_1.pdf]

Genomic *TCF12* (HEB) locus accession number:
NC_000075.6

HEBCan Annotated Protein Sequence:

HEBCan-specific exons

AD1 (1-99)
MFSPPVNSGKTRPTTLGSSQFSGSGMNPQQQRMAAIGTDKELSDLLDFSAMDERGGTTSWGTSGQPSPSYD
AD3 (100-305)
SSRGFTDSPHYSDHLNDSRLGTHEGLSPTPFMNSNLIGKTSERGSFSLYSRDSGLSGCQSSLLRQDLGLGS

PAQLSSSGKPGTPYYSFSATSSRRRPLHDSVALDPLQAKKVRKVPPGLPSS

HEBAlt-specific exon

Alt exon (1-23)
MYCAYPVPGMGNNSLMYYYNGKT

Exons shared by HEBCan and HEBAlt
AD3
VYAPSPNSDDFNRESPSYPSPKPPTSMFASTFFMQDGTHSSSDLWSSSNGMSQPGFGGILGTSTSHMSQSS
AD2 (306-548)
SYGSLHSHDRLSYPPHSVSPTDINTSLPPMSSFHRGSTSSSPYVAASHTPPINGSDSILGTRGNAAGSSQT

GDALGKALASIYSPDHTSSSFPSNPSTPVGSPSPLTGTSQWPRAGGQAPSSPSYENSLHSLQSRMEDRLDR

LDDAIHVLRNHAVGPSTSLPTSHSDIHSLLGPSHNASIGNLNSNYGGSSLVTNSRSASMVGTHREDSVSLN

GNHSVLSSTVAASNTELNHKTPENFRGGVQNQSGSVVPTEIKTENKEKDENLHEPPSSDDMKSDDESSQKD
bHLH (557-682)
IKVSSRGRTSSTNEDEDLNPEQKIER**EKERRMANNARERLRVRDINEAFKELGRMCQLHLKSEKPQTKLLI**

LHQAVAVILSLEQQVRERNLNPKAACLKRREEEKVSAASAEPPNTLPGAHPGLSESTNPMGHL

Fig. S1. Annotated amino acid sequence of HEB showing domain structures. Purple = activation domain 1 (AD1), light blue = activation domain 3 (AD3), pink = Alt exon, green = activation domain 2 (AD2), orange = basic helix-loop-helix (bHLH) DNA binding and dimerization domain. The amino acid numbers designated above each domain is based on the amino acid sequence of HEBCan exons, except for the Alt domain. Note that only the second half of the AD3 domain is contained within HEBAlt, and that the Alt exon is not contained within HEBCan.

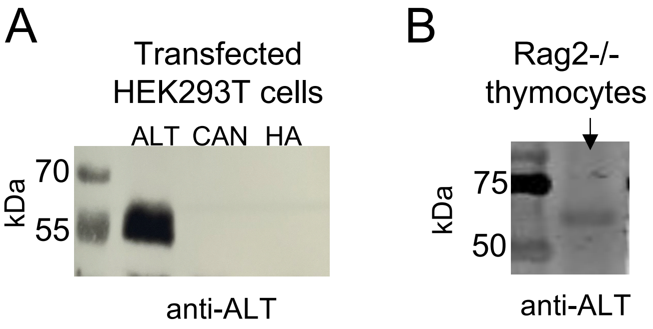

Fig. S2. Validation of the specificity of a rabbit-derived antibody that detects a peptide within the Alt domain of HEBAlt. HEK293T cells were transfected with constructs expressing HEBAlt (ALT), HEBCan (CAN) or empty vector (HA). Anti-ALT was used to detect (A) overexpressed HEBAlt in HEK293T cells or (B) endogenous HEBAlt in Rag2-/- thymocytes using a secondary rat anti-rabbit antibody conjugated to horseradish peroxidase, followed by visualization using chemiluminescence.
